# Unusual Presentation of Pulmonary Hydatidosis Mimicking Thoracic Malignancy in a Paediatric South African Patient

**DOI:** 10.5334/jbsr.1538

**Published:** 2018-10-26

**Authors:** Charlotte De Wilde, Anne-Marie du Plessis, Pierre Goussard

**Affiliations:** 1 Department of Radiology, Ghent University Hospital, Ghent University, Ghent, BE; 2 Division of Radiodiagnosis, Department of Medical Imaging and Clinical Oncology, Faculty of Medicine and Health Sciences, Tygerberg Hospital and Stellenbosch University, Cape Town, ZA; 3 Department of Paediatrics and Child Health, Faculty of Medicine and Health Sciences, Tygerberg Hospital and Stellenbosch University, Cape Town, ZA

**Keywords:** Thoracic mass, paediatric, hydatid, Echinococcus, pleuropulmonary blastoma, pulmonary malignancy

## Abstract

In this case report, an illustrative case of pulmonary hydatidosis in a young South African girl is presented. The acute symptomatology, rapidly worsening clinical condition and the extremely atypical appearance of the hydatid cysts on imaging were initially misleading and raised suspicion for malignant disease.

## Introduction

Hydatidosis is a parasitic disease caused by Echinococcus granulosus or Echinococcus multilocularis, endemic in South Africa [1]. The eggs of the parasite are passed on via dogs and sheep to find an accidental human host. Once ingested, the egg finds its way to the hepatic capillary bed, evolves into an embryo, and can either form cysts or spread hematogeneously [2].

Infected adults present in 75% of the cases with lesions in the liver, while less than 30% have pulmonary cysts. In the paediatric population, however, over 60% of patients present with pulmonary hydatidosis, and less than 10% have concomitant hepatic disease [3]. Pulmonary hydatid cysts in children are usually large at diagnosis, due to increased compressibility of the organ, allowing the cysts to grow faster. The cysts are usually spherical, solitary, and unilocular, with or without intralesional daughter cysts. Multiple cysts only occur in 30% of cases and bilateral cysts occur very rarely [2,3]. Pulmonary hydatidosis in children is most frequently caused by E. granulosus. There is, however, a morphological difference between the cysts caused by E. granulosus and E. multilocularis, the latter causing more multilocular and infiltrative cysts, without a pericyst [4].

This case report illustrates a very unusual appearance of pulmonary hydatidosis, with innumerable and bilateral cysts of different sizes, which – to our knowledge – has never been reported on before in paediatric patients.

## Case Report

**Figure 1:**
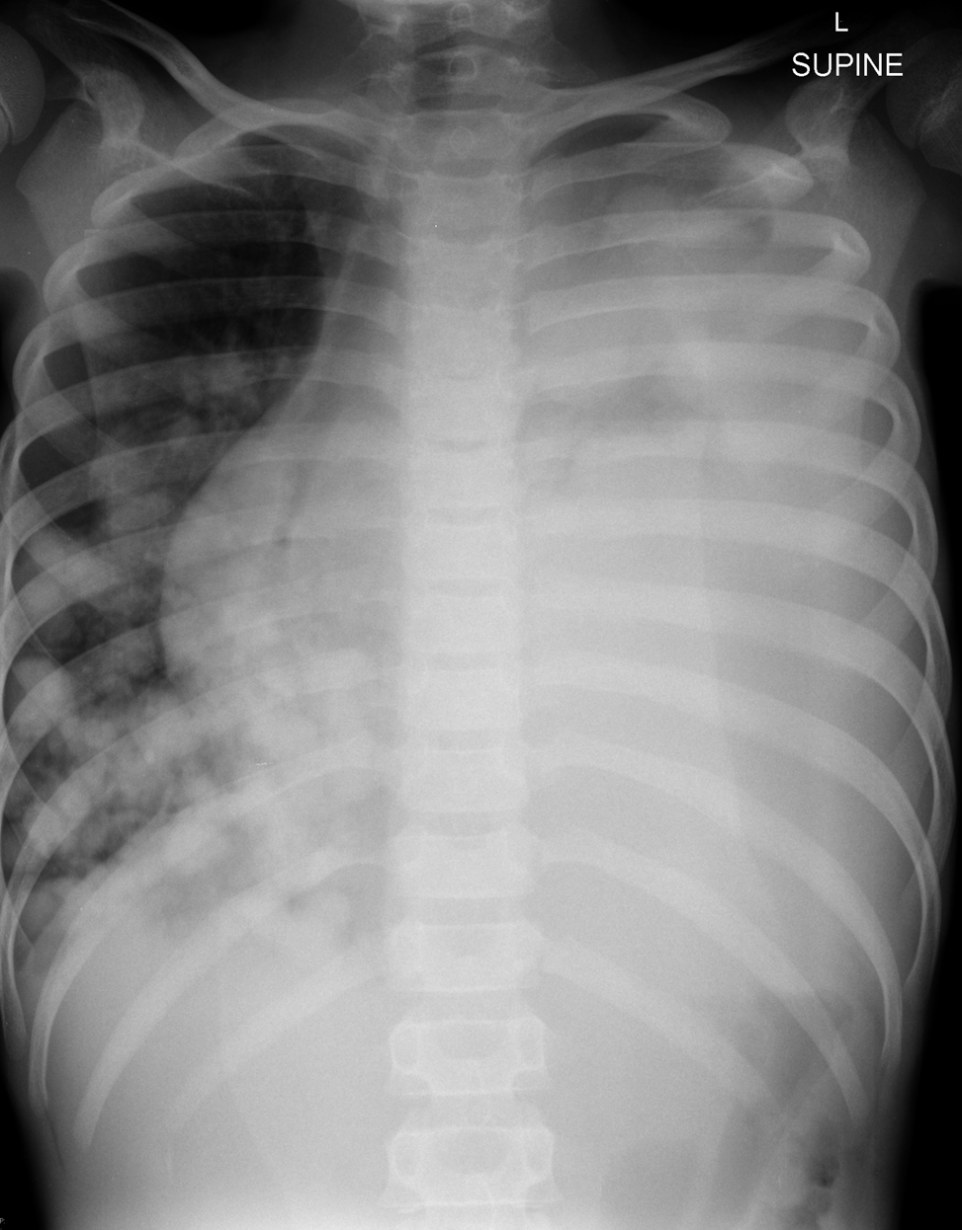
Chest x-ray of a 10-year-old patient presenting with respiratory distress, productive cough, and pleuritic chest pain shows complete opacification of the left lung and multiple round opacities in the right middle and lower lung fields.

**Figure 2:**
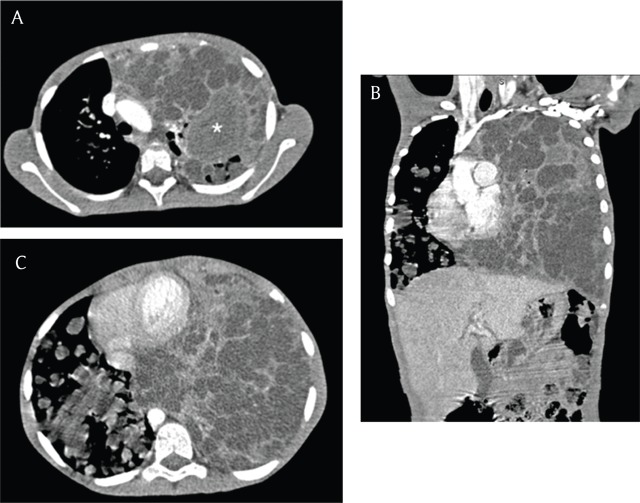
Axial and sagittal CT scan shows multiple round, cystic lesions, interspersed by atelectatic lung parenchyma on the right and fewer cystic lesions in the left lung with a residual moderate amount of aerated lung. There was one complicated cyst in the left upper lobe (Figure. 2A, asterisk). The liver shows no involvement.

We present an unusual case of pulmonary hydatid cysts mimicking malignancy in a 10-year-old girl. A paediatric, female patient presented with acute respiratory distress, productive cough, and pleuritic chest pain. The first-line investigations included a biochemical work-up and a chest X-ray. Laboratory results showed raised C-reactive protein and leucocytosis. The supine plain radiography showed complete opacification of the left lung with widening of the intercostal spaces, right-sided cardiomediastinal shift and multiple round opacities in the right middle and lower lung fields (Figure 1). A contrast-enhanced computed tomography (CT) scan (40-slice Siemens, CT Dose Index 4.25) demonstrated multiple densely packed cystic lesions, interspersed by atelectatic lung parenchyma on the left and multiple scattered cysts in the right lung, with a residual moderate amount of aerated lung. A spherical, soft tissue density lesion was seen in the left upper lobe. The liver demonstrated no involvement (Figure 2A–C).

Pulmonary malignancy, although seldomly encountered in this age group, was suspected due to the diffuse involvement of the lungs by the lesions and the soft tissue density lesion in the left upper lobe. Possible differentials included pleuropulmonary blastoma (especially type II), malignant teratoma, undifferentiated sarcoma, and diffuse pulmonary metastases. The most plausible differential was pleuropulmonary blastoma (PPB), an embryonal mesenchymal neoplasm and one of the most common primary lung malignancies in children. Type II PPB characteristically presents with both solid and cystic lesions of the lung and pleura and can be solitary or multiple [5]. Superinfection of the normally air-containing cysts in PPB was thought to explain the fluid content of the cysts. An atypical presentation of pulmonary hydatidosis was initially considered as a differential, but much less likely due to the presence of what appeared to be a soft tissue lesion in the left upper lobe.

**Figure 3:**
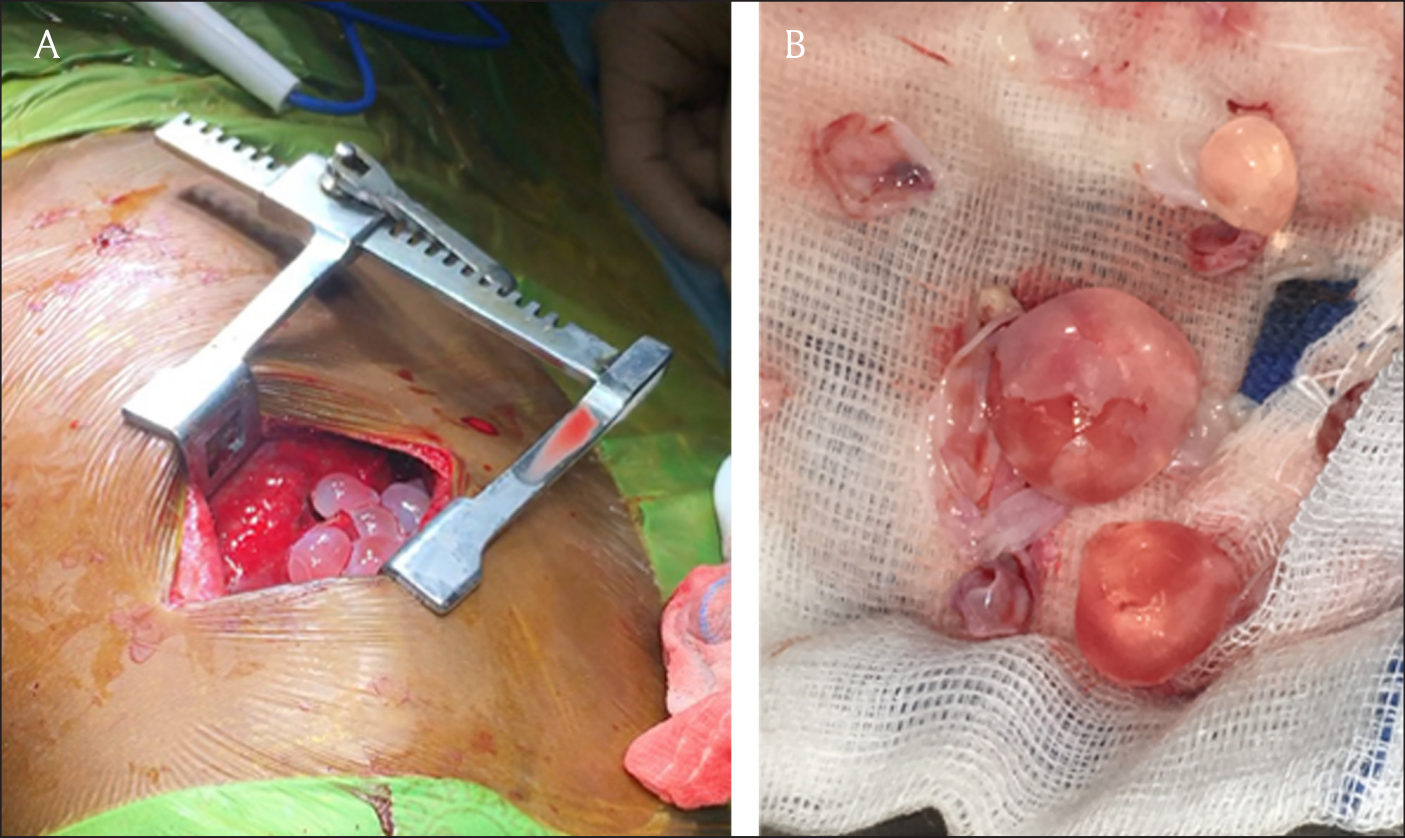
Peroperative photographs shows echinococcus cysts with several layers.

Open biopsy was performed, and multiple parenchymal hydatid cysts with innumerable daughter cysts were seen (Figure 3A–B). Serology of the patient and a positive antigen test (specific for Echinococcus granulosus) on the cyst fluid confirmed the diagnosis of pulmonary hydatidosis. A large amount of cysts was surgically removed during thoracotomy. Despite release of the cyst fluid into the circulation, neither anaphylaxis nor other complication occurred during and after the operation. The patient was then treated medically with albendazole and improved significantly. Following the confirmation of pulmonary hydatidosis, the apparent soft tissue density lesion in the left upper lobe, as visualized on CT, could be identified as a complicated cyst due to super-infection and haemorrhage – not a solid mass as initially suspected. This diagnosis was confirmed on the follow-up MRI, but the causative infectious agent could not be isolated.

**Figure 4:**
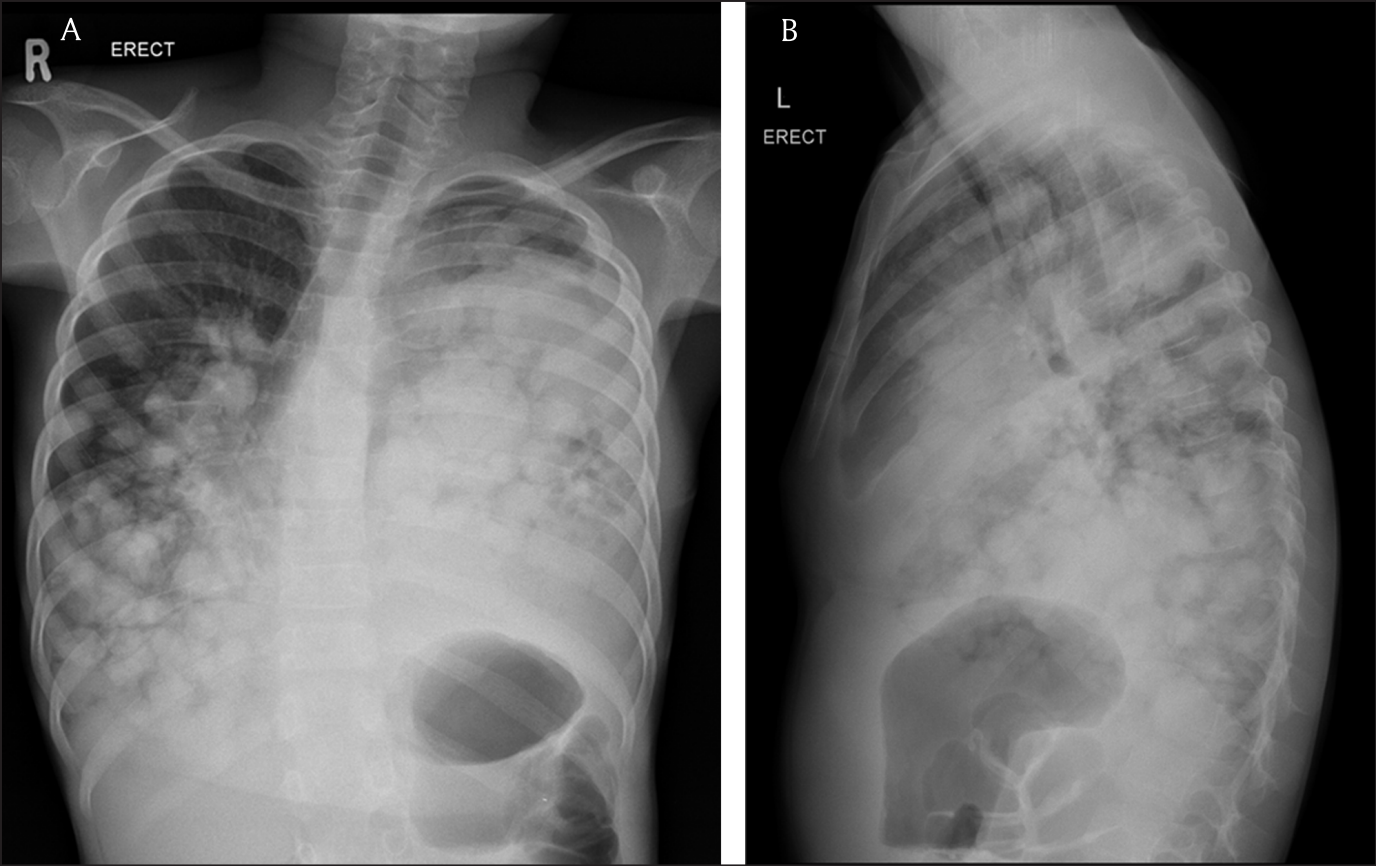
Follow-up frontal and lateral radiographs two weeks later showed interval decrease of the parenchymal hydatid cysts, especially on the left side.

**Figure 5:**
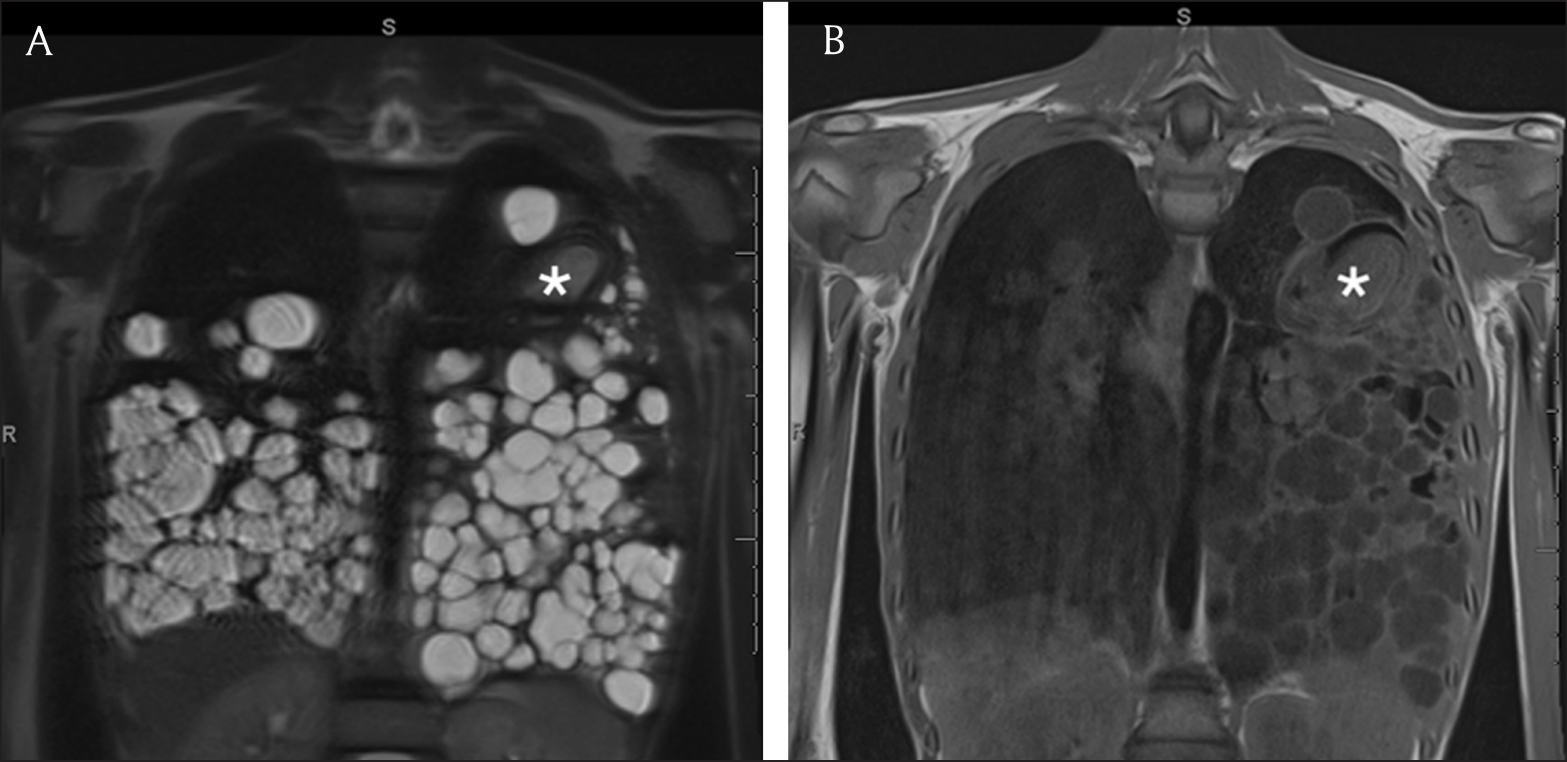
Coronal T2- and T1-weighted images of the chest demonstrate innumerable unilocular cysts with simple fluid content. One cyst in the left upper lobe (asterisk) has a more complex architecture and contains collapsed parasitic membranes.

Follow-up chest radiograph (Figure 4A–B) two weeks later and magnetic resonance imaging (MRI) of the chest six weeks after surgery were performed to evaluate the amount of remaining cysts. A decrease of the innumerable bilateral cysts was demonstrated. On MRI, almost all of them exhibited a hyperintense signal on T2WI and a hypointense signal on T1WI reflecting simple fluid contents – except one lesion in the left upper lobe, representing the complicated hydatid cyst (Figure 5A–B).

## Discussion

CT is the imaging modality of choice in hydatid disease and typically shows an uncomplicated cyst with a high-attenuation wall and a low density content, representing fluid. It is often possible to distinguish the pericyst and daughter vesicles. Death of the parasite is followed by calcification of the cyst. The lungs are the most common site of hydatid disease in the paediatric population and hydatid cysts in children are usually large at diagnosis. Multiple pulmonary cysts occur in 30% of the cases and bilateral cysts occur very rarely [3,6]. Apart from the pulmonary involvement without concomitant hepatic disease, our case is not, to our knowledge, the typical representation of pulmonary hydatidosis in a paediatric patient. The atypical presentation of our case is illustrated by the innumerable and bilateral cysts, of which one was super-infected and hemorrhagic – the latter mimicking a soft tissue component and thus raising suspicion for malignancy. Primary pulmonary malignancy (especially pleuropulmonary blastoma) and diffuse pulmonary metastases were considered as differential diagnoses based on the imaging characteristics of the lesions on X-ray and CT. Open biopsy, serology of the patient, and a positive antigen test on the cyst fluid confirmed the diagnosis of the benign condition caused by E. granulosus.

In this patient, repetitive infection due to exposure to the same source of E. granulosus is thought to have caused the atypical appearance with bilateral and innumerable cysts of different sizes.

Surgical removal of multiple cysts and systemic treatment with albendazole resulted in patient recovery and a significant decrease of the amount of hydatid cysts at two- and six-week follow-ups.

Finally, primary pulmonary malignancies are very rare in the paediatric age group and awareness of diagnostic traps – such as complicated hydatid cysts mimicking a solid tumour – are emphasized in this case report so as to avoid unnecessary and potentially harmful treatment.
